# The biochemical subtype is a predictor for cognitive function in glutaric aciduria type 1: a national prospective follow-up study

**DOI:** 10.1038/s41598-021-98809-9

**Published:** 2021-09-29

**Authors:** E. M. Charlotte Märtner, Eva Thimm, Philipp Guder, Katharina A. Schiergens, Frank Rutsch, Sylvia Roloff, Iris Marquardt, Anibh M. Das, Peter Freisinger, Sarah C. Grünert, Johannes Krämer, Matthias R. Baumgartner, Skadi Beblo, Claudia Haase, Andrea Dieckmann, Martin Lindner, Andrea Näke, Georg F. Hoffmann, Chris Mühlhausen, Magdalena Walter, Sven F. Garbade, Esther M. Maier, Stefan Kölker, Nikolas Boy

**Affiliations:** 1 Division of Child Neurology and Metabolic Medicine, Centre for Child and Adolescent Medicine, University Hospital Heidelberg, Heidelberg, Germany; 2 Division of Experimental Paediatrics and Metabolism, Department of General Paediatrics, Neonatology and Paediatric Cardiology, University Children’s Hospital, Heinrich Heine University Düsseldorf, Düsseldorf, Germany; 3 Children’s Hospital, University Medical Center Hamburg-Eppendorf, Hamburg, Germany; 4 Dr. Von Hauner Children’s Hospital, Ludwig-Maximilians-University, Munich, Germany; 5 Department of General Paediatrics, Metabolic Diseases, University Children’s Hospital Muenster, Muenster, Germany; 6 Charité—Universitätsmedizin Berlin, corporate member of Freie Universität Berlin, Humboldt-Universität Zu Berlin, and Berlin Institute of Health, Center for Chronically Sick Children, Berlin, Germany; 7 Department of Child Neurology, Children’s Hospital Oldenburg, Oldenburg, Germany; 8 Department of Paediatrics, Paediatric Metabolic Medicine, Hannover Medical School, Hannover, Germany; 9 Children’s Hospital Reutlingen, Reutlingen, Germany; 10 Department of General Paediatrics, Adolescent Medicine and Neonatology, Medical Centre, University of Freiburg, Faculty of Medicine, Freiburg, Germany; 11 Department of Pediatric Neurology and Inborn Errors of Metabolism, Children’s Hospital, University of Ulm, Ulm, Germany; 12 Division of Metabolism and Children’s Research Centre, University Children’s Hospital Zurich, Zurich, Switzerland; 13 Department of Women and Child Health, Hospital for Children and Adolescents, Centre for Paediatric Research Leipzig (CPL), University Hospitals, University of Leipzig, Leipzig, Germany; 14 Department of Pediatrics, Helios Klinikum, Erfurt, Germany; 15 Centre for Inborn Metabolic Disorders, Department of Neuropediatrics, Jena University Hospital, Jena, Germany; 16 Division of Paediatric Neurology, University Children’s Hospital Frankfurt, Frankfurt, Germany; 17 Children’s Hospital Carl Gustav Carus, Technical University, Dresden, Germany; 18 Department of Pediatrics and Adolescent Medicine, University Medical Center, Göttingen, Germany

**Keywords:** Metabolic disorders, Paediatric research

## Abstract

The aim of the study was a systematic evaluation of cognitive development in individuals with glutaric aciduria type 1 (GA1), a rare neurometabolic disorder, identified by newborn screening in Germany. This national, prospective, observational, multi-centre study includes 107 individuals with confirmed GA1 identified by newborn screening between 1999 and 2020 in Germany. Clinical status, development, and IQ were assessed using standardized tests. Impact of interventional and non-interventional parameters on cognitive outcome was evaluated. The majority of tested individuals (n = 72) showed stable IQ values with age (n = 56 with IQ test; median test age 11 years) but a significantly lower performance (median [IQR] IQ 87 [78–98]) than in general population, particularly in individuals with a biochemical high excreter phenotype (84 [75–96]) compared to the low excreter group (98 [92–105]; p = 0.0164). For all patients, IQ results were homogenous on subscale levels. Sex, clinical motor phenotype and quality of metabolic treatment had no impact on cognitive functions. Long-term neurologic outcome in GA1 involves both motor and cognitive functions. The biochemical high excreter phenotype is the major risk factor for cognitive impairment while cognitive functions do not appear to be impacted by current therapy and striatal damage. These findings implicate the necessity of new treatment concepts.

## Introduction

Glutaric aciduria type 1 (GA1; OMIM #231670) is a rare neurometabolic disorder of L-lysine metabolism caused by inherited deficiency of glutaryl-CoA dehydrogenase (GCDH) resulting in accumulation of glutaric acid (GA), 3-hydroxyglutaric acid and glutarylcarnitine especially within the brain. The *GCDH* gene is located on chromosome 19p13.2 encoding a flavin adenine dinucleotide dependent mitochondrial matrix protein catalysing the oxidative decarboxylation of glutaryl-CoA to crotonyl-CoA in the degradative pathway of L-lysine, L-hydroxylysine, and L-tryptophan. The estimated prevalence in Germany is 1:124.930^1^. Untreated individuals mostly develop striatal injury between the age 3–36 months which leads to a complex movement disorder (MD) with predominant dystonia due to bilateral striatal damage, manifesting *acutely* after acute encephalopathic crisis during potentially catabolic episodes such as febrile illness associated with high morbidity and mortality or *insidiously* without an apparent crisis^2,3^. Due to the primarily neurologic phenotype GA1 has been classified as a ‘cerebral organic aciduria’. Recently, however, also extra-neurological manifestations such as disturbance of kidney function have been reported^1,4^. Two biochemical subtypes in GA1, i.e., high excreter (HE) and low excreter (LE) have been arbitrarily defined depending on urinary concentrations of GA^5^, which is inversely correlated with residual enzymatic GCDH activity. Individuals with HE and LE subtype are thought to have a similarly high risk of striatal damage during infancy and the same a priori risk of developing a MD^2,6^. More recently, however, increased in vivo cerebral GA concentrations and progressive white matter abnormalities have been reported in HE patients, a finding of uncertain clinical relevance^7^. Metabolic treatment concepts have been developed during the last decades consisting of a low lysine diet with supplementation of a lysine-free, tryptophan-reduced, arginine-containing amino acid mixture and carnitine supplementation for maintenance treatment (MT) and transient emergency treatment (ET) during potentially catabolic episodes such as febrile illness or surgery. Implementation of GA1 into national newborn screening (NBS) programs facilitating early diagnosis as well as development and repetitive revision of evidence-based guideline recommendations have dramatically improved neurologic long-term outcome worldwide resulting in more than 90% asymptomatic individuals with GA1 without MD if treated in full accordance with the recommendations^1,8–13^.

In contrast to the significant motor phenotype, the clinical course in GA1 is not thought to typically include cognitive impairment^14^, but this has not been systematically studied before. Therefore, this study aims at prospectively investigating long-term cognitive development and functions of early diagnosed and treated individuals with GA1 in Germany over a period of over 20 years.

## Methods

### Study population

This national prospective multicentre observational study includes patients diagnosed with GA1 in Germany between January 1st, 1999, and February 29th, 2020. The study population comprised three subgroups: (1) individuals with GA1 correctly identified by NBS, (2) patients with false-negative NBS result identified by targeted metabolic work-up following the manifestation of characteristic neurological signs, and (3) previously undiagnosed women with GA1 identified by initially positive NBS results of their unaffected children (maternal GA1). Inclusion criteria comprised (1) confirmation of diagnosis by quantitative analysis of organic acids in urine (GA and 3-hydroxyglutaric acid) and/or *GCDH* mutation analysis and/or quantitative analysis of GCDH residual activity, and (2) written informed consent from patients and/or their parents.

### Cognitive outcome

Development and cognition were evaluated using age-dependent standardized developmental and intelligence tests, performed by trained psychologists and medical staff. For patients up to age three years the Denver Developmental Screening Test and cognitive scale of Bayley Scales of Infant Development II or III were used. The Denver Developmental Screening Test categorizes development in four scales as ‘normal’ or ‘abnormal’ (personal-social, fine motor-adaptive, language, and gross motor)^15^. The results of Bayley Scales of Infant Development are numeric and distributed normally with a mean of 100 and SD of 15^16^. Patients aged three years up to adulthood were tested with IQ tests developed by David Wechsler (Wechsler Preschool and Primary Scale of Intelligence [HAWIVA-III, WPPSI-III/-IV], Wechsler Intelligence Scale for Children [HAWIK-IV, WISC-IV/-V], Wechsler Adult Intelligence Scale [WAIS-IV]), Nadeen and Alan Kaufmann (Kaufmann Assessment Battery for Children [K-ABC/K-ABC-II]), and Anna Snijders-Oomen (Snijders-Oomen Nonverbal Intelligence Test [SON-R 2.5–7]). Full scale and subscale IQ results, all normalized to mean 100 with SD 15, were used for analysis. Subscales of different IQ tests were attributed to cognitive factors according to the Cattel-Horn-Carroll theory (Table 1)^17^. For a cross-sectional analysis, the most recent test result of each patient was used. For individuals with more than one IQ test an additional longitudinal analysis was performed.

**Table 1:** Attribution of IQ test subscales to cognitive functions (according to Cattell-Horn-Carroll [CHC] theory)^17^.

CHC-stratum II-factors^17^	WPPSI-III, HAWIVA-III	WPPSI–IV, WISC-V	WISC-IV, WAIS-IV	K-ABC II	SON-R	K-ABC*
Fluid reasoning	Performance (from 4.0 years)	Fluid reasoning	Perceptual reasoning	Planning ability (Fluid reasoning)	Reasoning scale	NA
Crystallised intelligence	Verbal	Verbal compre-hension	Verbal compre-hension	Knowledge (Crystallised ability)	NA	NA
Visual processing	Performance	Visual spatial	Perceptual reasoning	Simultaneous processing (Visual processing)	Performance scale	NA
Short-term memory	NA	Working memory	Working memory	Sequential processing (Short-term memory)	NA	NA
Processing speed	Processing speed (from 4.0 years)	Processing speed	Processing speed	NA	NA	NA

*CHC* Cattell-Horn-Carroll, *HAWIVA* Hannover-Wechsler-Intelligenztest für das Vorschulalter, *K-ABC* Kaufmann Assessment Battery for Children, *NA* not applicable, *SON-R* Snijders-Oomen Nonverbal Intelligence Test Revised, *WAIS* Wechsler Adult Intelligence Scale, *WISC* Wechsler Intelligence Scale for Children, *WPPSI* Wechsler Preschool and Primary Scale of Intelligence.

*Attribution was not possible.

### Biochemical subtype

According to a previous definition based on residual enzymatic GCDH activity and urinary GA concentrations, the biochemical subtype was arbitrarily classified as HE if GA concentration in urine was higher than 100 mmol/mol creatinine, or as LE if GA was equal to or below 100 mmol/mol creatinine^5^. Further definitions of biochemical subtypes using clinical variables have not been established for GA1 due to the similar clinical course of both subgroups. For additional analysis, we delineated an intermediate biochemical subgroup of patients with initially elevated GA concentrations of 100–1000 mmol/mol creatinine rapidly declining below 100 mmol/mol creatinine after the start of metabolic treatment. Only those individuals whose GA concentrations remained above 100 mmol/mol GA were classified as HE.

### Treatment

According to the current guideline recommendations^9^, metabolic MT consists of a (1) low-lysine diet (i.e. age- and weight-dependent calculated reduction of daily lysine intake) with supplementation of a lysine-free, tryptophan-reduced, arginine-fortified amino acid supplement maintaining a sufficient energy and micronutrient intake for patients up to age six years, (2) a liberalized protein-controlled diet for patients beyond age six years, and (3) life-long oral carnitine supplementation. Intermittent ET is recommended in every potentially catabolic situation and consists of transiently (1) high carbohydrate intake, (2) low to no protein intake, and (3) intensified carnitine supplementation. Treatment was assessed as adequate only if guideline recommendations were followed throughout the patient’s course. Patients were classified as being supervised by a metabolic centre if supervision had started during the neonatal period and continued until the last follow-up visit.

### Neurologic manifestations

Based on the patients’ medical history and clinical examination, neurologic manifestations were classified as (1) *major* if patients developed MD, (2) *minor* if patients had fine motor or coordination deficits in the absence of MD, and (3) *asymptomatic* if none of these symptoms existed. MD was *mild* if it did not cause serious disability in daily life, *moderate* in the case of motor disability affecting daily life, but with some motor functions being preserved, and *severe* if only few or no motor skills were left. Onset-type of MD was assessed as (1) *acute* following encephalopathic crisis or subdural haemorrhage, or (2) *insidious* without apparent crisis.

### Kindergarten and school

Attendance of kindergarten and school was classified as (1) regular kindergarten/school, (2) regular kindergarten/school with remedial teaching, and (3) kindergarten/school for children with special educational needs.

### Statistical analysis

Statistical analysis was conducted using the statistical package R^18^. For all parameters, sample size, mean, median, SD, interquartile range (IQR), and range were calculated (Table 2). Independent variables for cognitive outcome analysis comprised (1) sex, (2) biochemical subtype, (3) neurologic manifestations, (4) adherence to recommended MT, and (5) adherence to recommended ET. Impact on full scale IQ and subscale results was investigated in a cross-sectional and a longitudinal analysis with multiple linear regression models and linear mixed effects models (R package lme4) using Satterthwaite’s method for estimating degrees of freedom^19^. Variable reduction in regression models was applied using a forward and backward stepwise procedure based on Akaike Information Criteria. We used classification and regression trees (R package rpart)^20^ analysis to identify subgroups with different IQ according to independent variables. Post-hoc comparisons in multiple regression models and classification and regression trees were applied with Tukey honestly significant difference method. We present data as boxplots which show IQR (box), median (horizontal line in box), and mean (triangle in box). Whiskers extend to the most extreme data point which is no more than 1.5 times the length of the box away from the box; points outside the range of whiskers can be considered as outliers.

**Table 2:** Cognitive outcome: development and full scale IQ of individuals identified by NBS.

A: Denver Developmental Screening Test						B: Bayley Scales of Infant Development			
	Total	Personal-social	Fine motor-adaptive	Language	Gross motor	Cognitive Scale	All patient (n = 19)	Biochemical subtype	
								HE (n = 14)	LE (n = 5)
Normal	17	20	20	19	17	Mean, SD Median [Q1, Q3] Min, Max p-value	91.37, 18.4 93 [83.5, 105] 49, 120	86.14, 18.34 88.5 [73.5, 96] 49, 120	106, 8.22 107 [106, 110] 103, 116 p = 0.0174
Abnormal	6 (+ 1 unclear)	3 (+ 1 unclear)	4	5	7				

C: IQ tests									
Full scale IQ	All patients	Sex		Neurologic abnormality					
		Female (n = 28)	Male (n = 28)	No (n = 34)	Minor (n = 7)	Major (n = 15)			
Mean, SD Median [Q1, Q3] Min, Max	86.61, 14.76 86.5 [77.75, 98.25] 58, 113	87.61, 15.16 87.5 [78.75, 98.25] 60, 113	85.61, 14.55 86 [75.25, 97.5] 58, 112	89.47, 13.17 87.5 [81, 100.5] 60, 113	78, 16.68 78 [65.5, 89] 58, 101	84.13, 16.25 89 [69, 96] 59, 112			
p-value		p = 0.9048		p = 0.5146					

Full scale IQ	Biochemical subtype		Maintenance treatment		Emergency treatment				
	HE (n = 42)	LE (n = 12)	No (n = 13)	Yes (n = 43)	No (n = 6)	Yes (n = 50)			
Mean, SD Median [Q1, Q3] Min, Max	84.33, 14.41 83.5 [75.25, 96.25] 58, 112	95.92, 13.71 98 [91.5, 104.75] 61, 113	83.46, 16.17 84 [75, 95] 59, 112	87.56, 14.37 87 [79.5, 99] 58, 113	80, 16.43 77 [68, 93.5] 61, 101	87.4, 14.52 87.5 [79.25, 98.75] 58, 113			
p-value	p = 0.164		p = 0.9515		p = 0.9589				

(A) Most patients assessed with Denver Developmental Screening Test showed normal development. (B) Median results in Bayley Scales of Infant Development were normal with HE patients showing lower results than LE patients. (C) Analysis of full scale IQ: Median results of all patients were in the lower average range, while patients with biochemical HE phenotype tended to have lower results than patients with LE phenotype.

*HE* high excreter, *LE* low excreter, *Max* Maximum, *Min* Minimum, *n* number of patients, *NBS* newborn screening, *Q1* 25th percentile, *Q3* 75th percentile.

### Standard protocol approvals, registrations, and patient consents

The study was approved by the Institutional Ethics Committee of the coordinating centre (University Hospital Heidelberg, application no. S-525/2010) as well as all contributing study sites. All methods were performed in accordance with relevant guidelines and regulations. All patients and/or their parents gave written informed consent to participate in the study and to be included into the European registry and network for Intoxication type Metabolic Diseases (https://www.eimd-registry.org/)^21^.

## Results

### Study population and epidemiology

The study sample included 107 patients with confirmed GA1, 98 of whom were identified by NBS, while six patients were missed by NBS and three maternal patients were diagnosed due to positive NBS results of their unaffected offspring. According to the *German Society of Newborn Screening* (https://www.screening-dgns.de/), the study cohort comprised 97.2% (69 of 71) of individuals identified by the German NBS program between 2004 and 2017 (so far, no data have been reported for the time before and after this interval). Overall sensitivity of NBS during the study period was 94.2%, but differed between HE (n = 78, 100%) and LE patients (n = 18, 75%; n = 2 patients with unknown biochemical subtype). Ninety-three patients (95%) of the NBS group survived until last visit and five patients (5%) died, four of whom with severe or moderate dystonic MD, as previously reported^1,22^.

In the NBS group, results of cognitive tests were available for 72 patients. Fifty-eight patients had at least one IQ test (multiple testing in 22 individuals) and 14 infants had developmental tests (Denver Developmental Screening Test: n = 13, Bayley Scales of Infant Development: n = 3; multiple tests possible). Reasons for missing (IQ) tests comprised rejection by patients or parents (n = 12), non-acceptance of the offer by the metabolic centre (n = 4), logistic problems (n = 6), severity of MD (n = 6), death (n = 5), and young age not allowing application of IQ tests (n = 7).

Tested individuals in the NBS group (39 females, 33 males) were diagnosed at a median (IQR, range) age of seven days (6–9, 2–84) and were tested at a median (IQR, range) age of 7.9 years (4.9–12.4, 0.7–19.1), with a cumulative follow-up time of 651.3 years. In 65 patients, positive NBS results were confirmed by *GCDH* mutation analysis and/or quantitative analysis of GCDH residual activity, and seven patients were diagnosed by quantitative analysis of organic acids in urine (GA and 3-hydroxyglutaric acid). Fifty-six patients (78%) were classified as HE subtype and 14 patients (19%) as LE subtype according to the previous definition^5^. A subgroup of 11 HE patients, in whom urinary GA excretion decreased below 100 mmol/mol creatinine after the start of metabolic therapy, was considered as intermediate subtype for additional analysis. The biochemical subtype remained unknown in two patients. Median (IQR) urinary GA concentration at last visit was 980 (554–1908) mmol/mol creatinine in HE patients, 7.5 (1.7–26) in intermediate patients, and 4 (0.6–10.3) in LE patients. Sixty-two patients (86%) attended kindergarten, 56 (90%) of whom attended regular kindergarten and six patients (10%; 4 of 6 with MD) attended kindergarten for special needs. Fifty-two patients (72%) attended school, 31 (60%) regular school (n = 5 with MD, n = 26 asymptomatic), while four patients (8%, 3 of 4 with MD) had additional remedial teaching in a regular school, and 13 patients (25%) attended a school for special education needs (n = 7 with MD, n = 3 with minor neurologic symptoms, n = 3 without motor symptoms). Three adult patients (6%) had successfully finished regular school (2 of 3 with MD), and the school career of one patient was unknown.

### Clinical outcome

None of the NBS patients showed signs of MD at diagnosis. At last visit, motor functions were normal in 43 of 72 patients with cognitive tests (60%), while seven patients (10%) had minor neurologic abnormalities and 22 patients (31%) had complex MD, mostly dystonia (n = 21). Dystonia was usually mild (n = 11; 52%) or moderate (n = 8; 38%), while two patients (10%) developed severe dystonia. Onset-type of dystonia was *acute* after encephalopathic crisis (n = 5; 23%) or following minor head trauma with subsequent subdural haemorrhage (n = 2; 9%), and *insidious* in 15 patients (68%). In 11 patients, dystonia was accompanied by ataxia (n = 3), ataxia plus chorea (n = 2), ataxia plus spasticity (n = 2), chorea (n = 2) or spasticity (n = 1). Another symptomatic patient had isolated ataxia.

Fifty-three patients (74%) followed the guideline recommendations for MT, while 19 (26%) did not. Deviations from MT comprised non-adherence to treatment protocols (n = 8), prescribed treatment protocols deviating from current recommendations (n = 7), feeding problems (n = 3), and delayed start of treatment (n = 1). Full adherence to ET was found in 62 patients (86%), vs. non-adherence in n = 10 (14%). Sixty-two patients (86%) were followed by a paediatric metabolic centre, while ten patients (14%) were in care of local hospitals or resident paediatricians without metabolic focus.

### Cognitive outcome in the NBS cohort

Detailed information is summarized in Table 2 and Supplementary Table 1.

### Development in infants and young children (0–3 years)

Twenty-four patients were evaluated by Denver Developmental Screening Test. Median (IQR) age at last screening test was 2.4 (1.5–3.5) years. Fifteen patients (63%) showed normal development in all domains, whereas nine patients (27%, seven of whom had dystonic MD) showed isolated (n = 3), combined (n = 4) or global (n = 2) developmental delay (Table 2).

Test results of Bayley Scales of Infant Development were available for 19 patients at a median (IQR) test age of 2.2 (1.5–3) years. Results of the cognitive scale were in the lower average range (median [IQR] 93 [84–105]) and remained stable over time (p = 0.6883) but differed significantly between HE (n = 14; median [IQR] 89 [76–95]) and LE patients (n = 5; median [IQR] 107 [103–110]; p = 0.0174).

### Cognitive functions are impaired compared to the general population

Overall, the median (IQR) IQ of tested individuals with GA1 was 87 (78–98) at the last documented IQ test (median [IQR] age 11 [14.1–19.1] years). Median IQ (IQR) of patients (n = 29) attending regular school was 95 (84–102), 70 (68–78) in patients (n = 4) with remedial teaching in a regular school, and 70 (61–81) in patients (n = 10) attending a school for special educational needs.

### Biochemical HE phenotype is a risk factor for cognitive impairment

**Figure 1:**
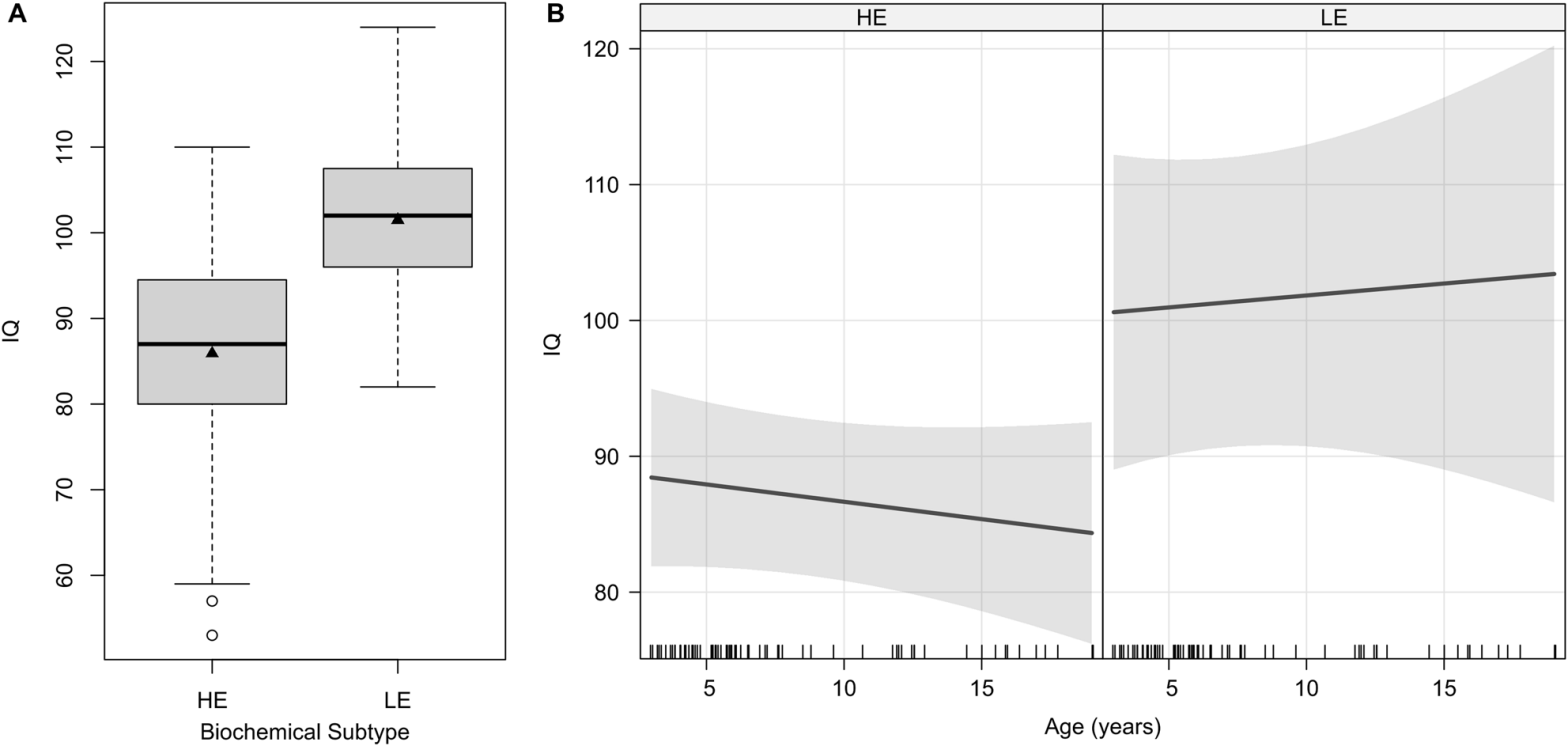
Biochemical subtype and full scale IQ at last visit (**A**) and over different age groups (**B**). (**A**) Patients with biochemical HE phenotype (median [horizontal line in box] 84; IQR 75–96) had lower full scale IQ than patients with biochemical LE phenotype (median [IQR] 98 [92–105]; p = 0.0164). (**B**) Longitudinal analysis was conducted using linear mixed model, gray area indicate 95% confidence interval bands. IQ was stable over time (p = 0.361), but, depending on the biochemical subtype, at different levels (p = 0.018). Rugs on X-axis indicate individual measurements. Black triangles indicate mean IQ. *HE* high excreter, *LE* low excreter.

In analogy to the test results of Bayley Scales of Infant Development, the biochemical subtype had an impact on full scale IQ with LE patients (n = 12; median [IQR] 98 [92–105]) showing superior results compared to HE patients at last visit (n = 42; 84 [75–96]; p = 0.0164; Fig. 1A). The effect was emphasized in a longitudinal analysis of 22 patients using a linear mixed model showing different levels depending on the biochemical subtype of about one SD (estimate 0.9 SD = 13.5 IQ points; LE [n = 6]: median [IQR] IQ 102 [97–107] vs. HE [n = 16]: 87 [78–94]; p = 0.018; Fig. 1B). Additionally, this analysis revealed stable cognitive performance over time (p = 0.361; Fig. 1B).

**Figure 2:**
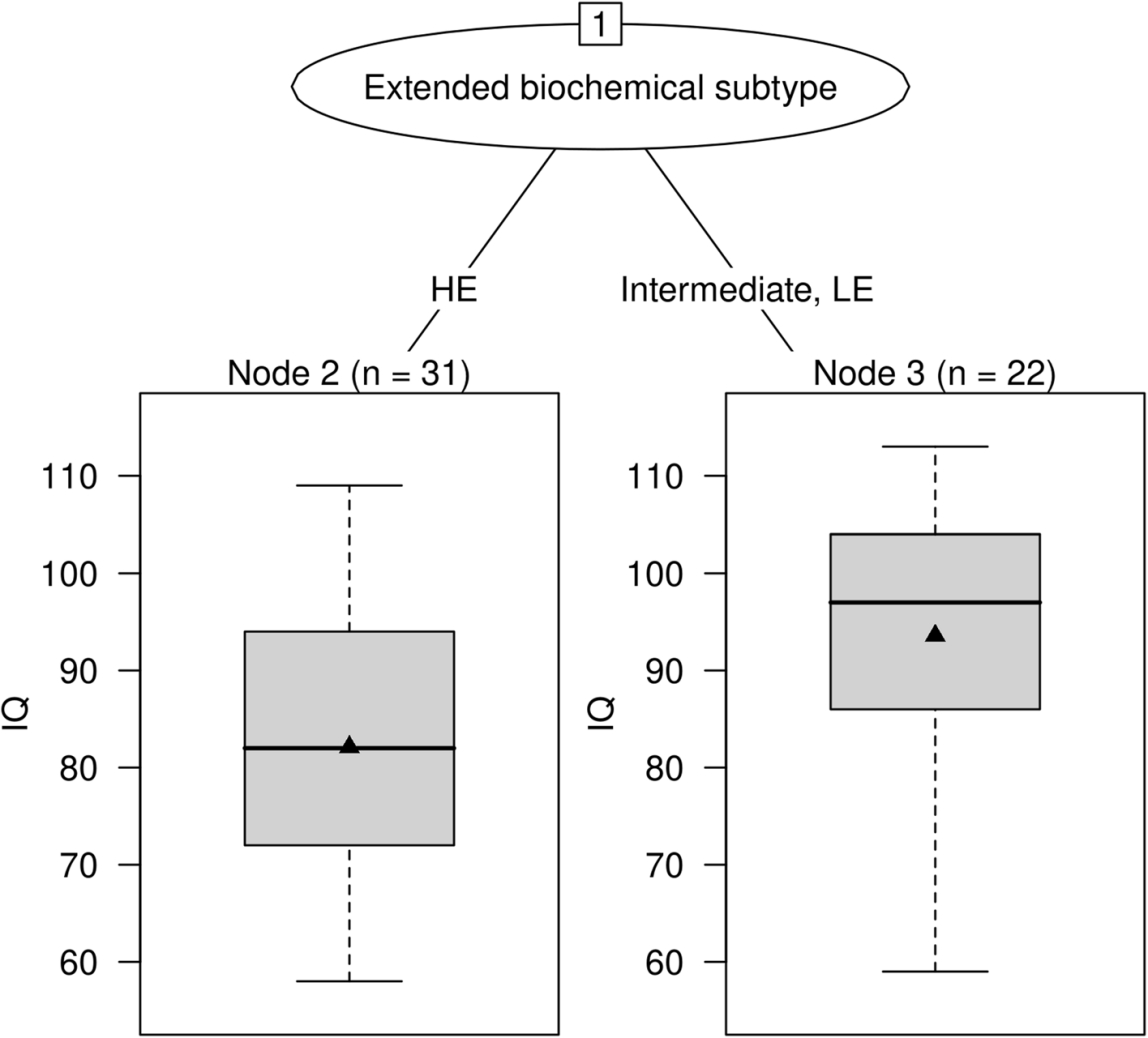
Extended biochemical subtype and full scale IQ. Patients with intermediate subtype had similar results as LE patients (p = 0.677). Results of these two groups (median [horizontal line in box] IQ 97; IQR 86–104) differed from HE patients (median [IQR] IQ 82 [72–94]; p = 0.005). Black triangles indicate mean IQ. *HE* high excreter, *LE* low excreter.

Next, we evaluated whether cognitive performance of the biochemical intermediate group differed from HE patients with constantly high GA concentrations or LE patients with constantly low GA concentrations. Patients assigned to the intermediate group (n = 10; median [IQR] IQ 92 [84–101]) showed a similar test performance as LE patients (n = 12; 98 [92–105]; p = 0.6771). Classification and regression trees analysis classified LE and intermediate patients as one group (n = 22; median [IQR] IQ 97 [86–104]) with higher performance level compared to HE patients (n = 31; 82 [72–94]; p = 0.0053; Fig. 2).

**Figure 3:**
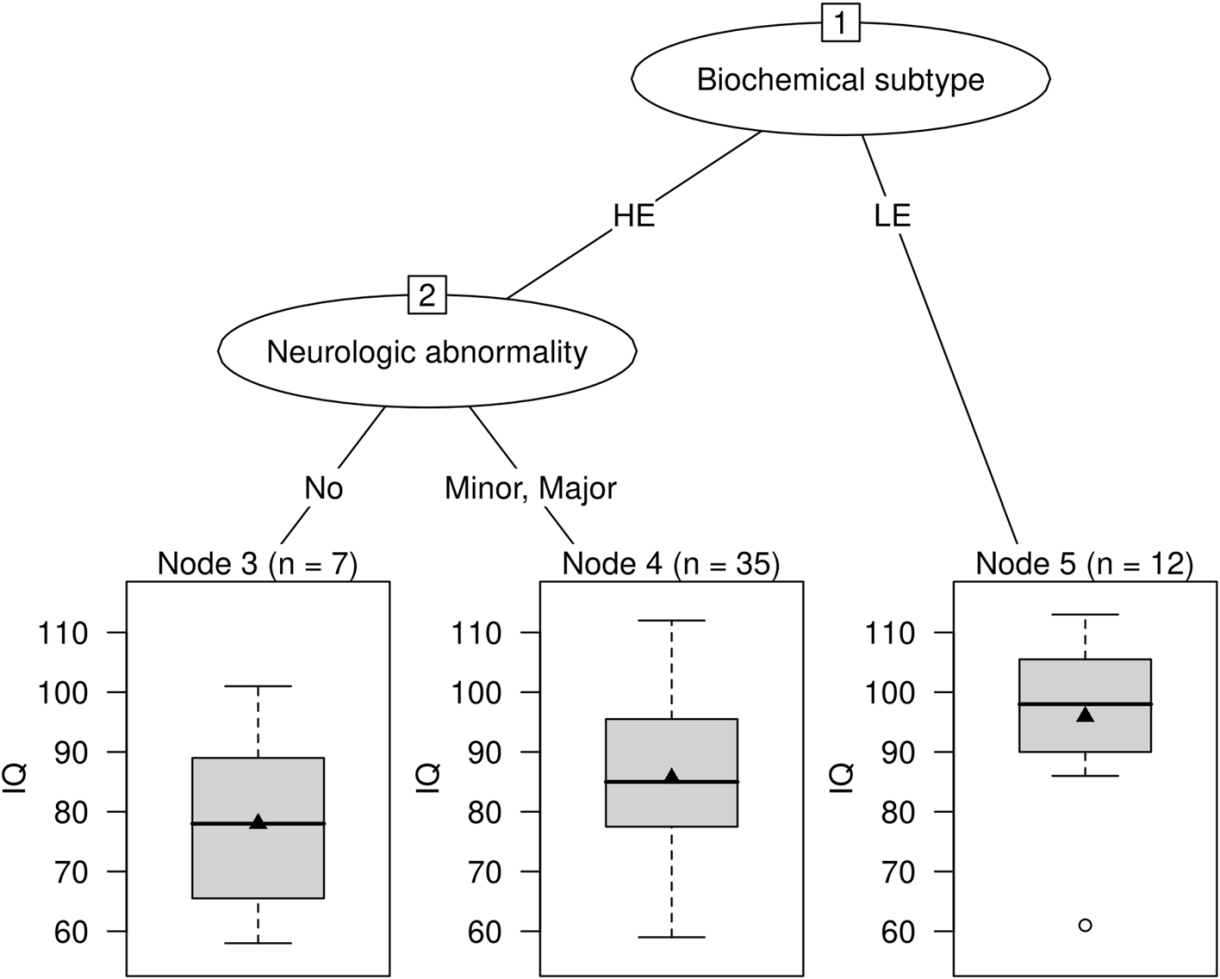
Biochemical subtype, neurologic abnormalities and full scale IQ. While biochemical subtype had a strong impact on full scale IQ, neurologic abnormalities did not. Patients with HE phenotype and minor neurologic abnormalities (median IQ [horizontal line in box] 78; IQR 66–89) had lower results than LE patients (median [IQR] IQ 98 [92–105]; p = 0.0278). Results of HE patients did not differ depending on neurologic abnormalities (p = 0.4045). Black triangles indicate mean IQ. *HE* high excreter, *LE* low excreter.

Motor phenotype and severity of MD did not have an impact on IQ. Median (IQR) IQ of patients with MD (n = 15) was 89 (69–96), while it was 78 (66–89) in patients with minor neurologic abnormalities (n = 7), and 88 (81–101) in asymptomatic patients (n = 34). Classification and regression trees analysis confirmed the strong impact of the biochemical subtype on IQ while cognitive functions of patients with and without neurologic abnormalities did not differ (p = 0.4045; Fig. 3).

### Treatment quality has no impact on cognitive functions

**Figure 4:**
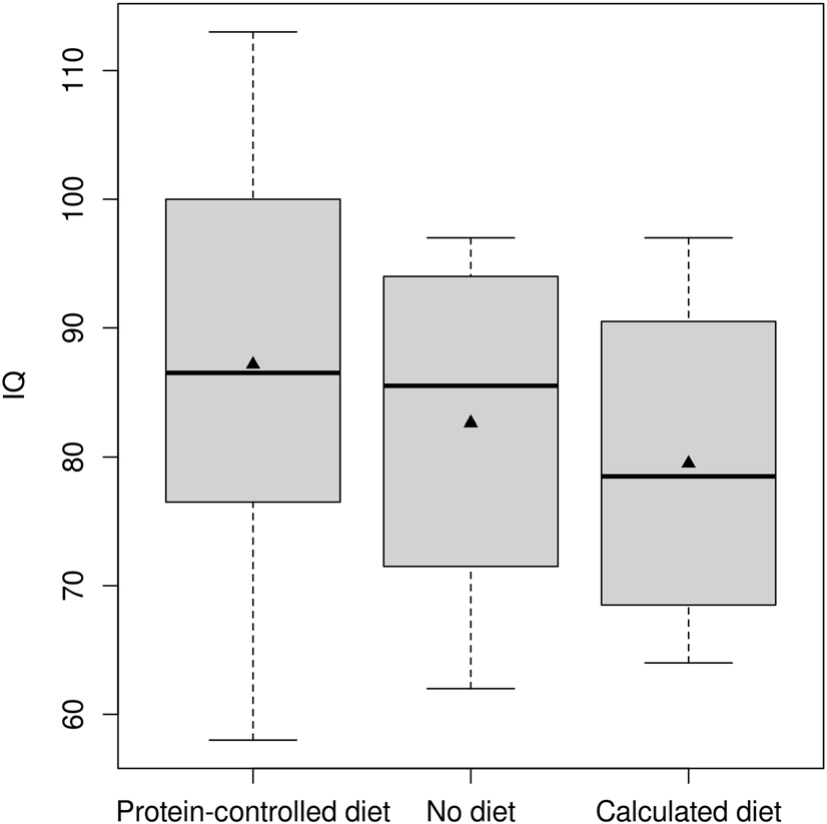
Maintenance treatment and full scale IQ in patients beyond age six years. Results did not differ between patients following recommended protein-controlled diet (median [horizontal line in box] IQ 87; IQR 77–100), patients continuing calculated diet beyond age six years (median [IQR] 79 [71–87]) and patients not complying to any diet (median [IQR] 86 [74–94]; p = 0.563). Black triangles indicate mean IQ.

Effects of metabolic treatment on neurologic outcome of the cohort were published separately^1^. Full scale IQ did not differ between patients with and without adherence to recommended MT (with MT [n = 43]: median [IQR] 87 [80–99] vs. without [n = 13]: 84 [75–95]; p = 0.942) and ET (with ET [n = 50]: 88 [79–99] vs. without [n = 6]: 77 [68–94]; p = 0.9728). In patients beyond age six years (n = 44; median [IQR] age at last test 11.9 [8.1–15.9] years), IQ did not differ between 32 patients following a protein-controlled diet according to the guideline recommendations (median [IQR] 87 [77–100]), eight patients no longer following a diet (86 [74–94]) and four patients still continuing a calculated diet (79 [71–87]; p = 0.563; Fig. 4).

### Subscale analysis

**Figure 5:**
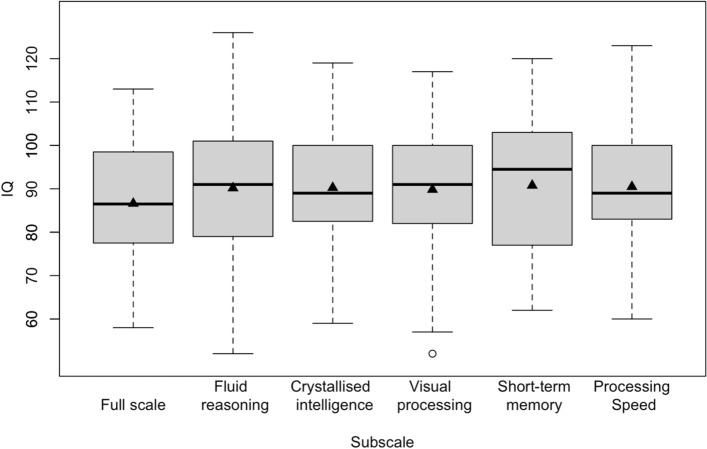
Subscale analysis. Results on subscale level were similar to full scale IQ and did not differ significantly (p = 0.7406). Black triangles indicate mean IQ.

Subscale analysis included 54 of 72 patients with an intelligence test, and complete datasets with results for full scale and all five subscales were available for 28 patients (Table 1, Supplement Table 1). Median (IQR) IQ results ranging from 89 (84–96) for crystallised intelligence to 96 (79–101) in short-term memory indicated homogenous performance on both subscale and full scale IQ levels (median [IQR] full scale IQ of this subset 89 [78–100]; fluid reasoning 95 [82–106]; visual processing 92 [82–102]; processing speed 92 [83–101]; p = 0.7406; Fig. 5). Analysing performance on subscale level for each biochemical subtype separately showed homogenous results for HE (p = 0.7184) and LE patients (p = 0.7676). However, results differed significantly depending on biochemical subtype for fluid reasoning (p = 0.0479), crystallised intelligence (p = 0.0433), short-term memory (p = 0.0689), and processing speed (p = 0.0047) with LE patients showing superior results compared to HE patients.

### Patients missed by NBS

All six patients missed by NBS (5 female, 1 male) belonged to the LE group, and five of them were diagnosed by targeted metabolic work-up after acute onset of MD (n = 3 with moderate, n = 2 with severe dystonia) while one asymptomatic patient was identified by high-risk family screening following diagnosis of an index patient. Numeric results of Bayley Scales of Infant Development and IQ tests ranging from 95 to 120 were similar to LE patients correctly identified by NBS.

### Maternal GA1 patients

Neurologic outcome of three maternal GA1 patients was reported previously^1^. Full scale IQ, available for two patients, was 82 and 106 respectively and was stable over time (79 and 82 in one patient). For another patient, only non-verbal tests could be performed, and results were in the lower average range or below-average (fluid reasoning/visual processing: IQ 72; processing speed: IQ 86).

## Discussion

The major findings of this study investigating the long-term cognitive outcome of individuals with GA1 identified through NBS in Germany between 1999 and 2020 are that (1) clinical phenotype of individuals with GA1 does include cognitive impairment despite NBS, (2) the biochemical HE subtype is the major risk factor for cognitive impairment, while early diagnosis by NBS, quality of treatment, and striatal damage do not have a measurable impact on cognitive functions, (3) cognitive functions are individually stable over time, and (4) full scale and subscale IQ results are homogenous.

### Deep phenotyping in GA1: Unravelling the long-term impact on cognitive functions

Research on rare diseases is also the history of scientific clinical endpoint evolution and precise exploration of the clinical phenotype. Early studies on GA1 focussed on acute manifestation of a complex MD with predominant dystonia, demonstrating a similarly high risk of striatal damage for untreated HE and LE individuals and a dramatic decline of encephalopathic crises in patients identified by NBS and early treated^23^. The phenotypic spectrum was extended by *insidious*-onset dystonia, which is still observed in screened individuals not adhering to or not receiving recommended MT^1,10^. Anthropometric and MRI studies elucidated phenotypic differences between the two biochemical phenotypes; HE individuals more often than LE individuals developed macrocephaly and progressive white matter abnormalities with unclear clinical relevance^24,25^. Recently, chronic kidney dysfunction, which was not impacted by recommended therapy or the neurologic phenotype, was identified as a first non-neurologic disease manifestation^1,4^, and malignant brain tumours and audiological manifestations were described in single patients^26,27^.

It had been hypothesized that the clinical phenotype of GA1 individuals does not typically include cognitive impairment regardless of severe dystonic MD^14^. Thirty years later, it is still unclear whether this hypothesis holds true. Earlier case reports and small case series supported the notion of unaffected cognitive functions in NBS patients^28–31^, and cross-sectional analysis of computer-based testing of developmental functions and cognitive performances showed similar results in GA1 patients and healthy controls while dystonic patients showed impairment in tests measuring motor speed, but not in tests with higher cognitive load^28^. In contrast, cognitive decline was described in adult patients^32^, and deficits in short- and long-term memory were recently demonstrated in Gcdh-deficient mice, an animal model for GA1 with complete loss of enzymatic GCDH activity^33^.

In this large prospectively followed cohort of early diagnosed individuals with GA1, we clearly demonstrate that neurologic long-term outcomes in GA1 do not only include significant motor dysfunction as previously reported^1,2^, but, as a new observation, also cognitive impairment independent from motor phenotype, found in one third (17/52) of early diagnosed patients requiring special education in school, and depicting an additional socio-economic burden. To identify affected patients requiring supportive treatment, the current guideline recommendations integrate developmental and IQ-assessment into the clinical long-term monitoring of GA1 patients^9^. We found stable intellectual performance over time in all age groups from early infancy to adulthood, and no significant correlation of the neurologic (motor) phenotype with cognition. Furthermore, we did not detect a ‘GA1-specific’ subscale pattern, e.g., cognitive performance was homogenous on subscale level, which is in line with a previous study^29^. This finding indicates that affected patients need a generalized and combined approach of supportive treatments and special education in kindergarten or school, and no subscale-specific approach. Consequently, full scale IQ is an appropriate tool for intellectual assessment in GA1, similarly to other intoxication type metabolic diseases, such as ornithine transcarbamylase deficiency^34^.

### The HE phenotype is the major risk factor for impaired cognitive outcome

Cognitive function and information processing require structural and functional integrity of cortex, basal ganglia, and thalamus^35^. Besides irreversible striatal damage found in symptomatic patients, GA1 patients are known to develop characteristic and highly dynamic MRI abnormalities (yet, of unclear clinical relevance) that may be present at birth, diminish under treatment (e.g., pallidal hyperintensity, frontotemporal hypoplasia) or progress with age (e.g., white matter abnormalities)^36^.

The two biochemical phenotypes in GA1 have been defined more than two decades ago^5^. Individuals with a HE subtype show a (virtually) complete loss of GCDH activity in contrast to up to 30% residual enzyme capacity found in LE subtype. The biochemical subtype is known to correlate with the genotype and residual enzyme activity, but *not* to predict the clinical phenotype^6,37^. Interestingly, patients with a hypothesized ‘intermediate’ biochemical subtype in our study showed similar cognitive results and long-term GA concentrations as individuals with LE subtype thus extending the range of initial biochemical abnormalities found in LE subtype as provided by a previous definition^5^.

However, recent MRI and ^1^H-magnetic resonance spectroscopy studies have observed discrepancies between HE and LE individuals, such as progressive periventricular white matter abnormalities in HE patients^7,24,36^, and progressive subependymal nodules in late diagnosed adults of the HE group, that had been explained by chronic neurotoxicity^1,24^. Lately, malignant brain tumours have been reported in three HE patients not receiving recommended therapy^27^. A recent study also reported on larger head circumferences in HE compared to LE patients^25^. In fact, the clinical impact of these observations is still unknown. Our study clearly demonstrates poorer cognitive outcome of individuals with HE phenotype compared to LE patients and the general population and thus, a first significant impact of the biochemical subtype on the clinical course. However, pathomechanism and aetiology of the diverse distribution of chronic neurotoxicity patterns among the two subtypes remain unclear. Accumulating GA and 3-hydroxyglutaric acid affect cellular functions on multiple levels impairing Krebs cycle enzymes^38^, interfering with anaplerotic transport processes between astrocytes and neurons^39^ as well as glutamatergic and GABAergic neurotransmission^40^, thus inducing brain energy impairment^38^, and generating increased amounts of reactive oxygen species^41,42^. Post-mortem studies showed massive accumulation of GA and 3-hydroxyglutaric acid^43^, which was explained by intracerebral de novo synthesis and entrapment of toxic metabolites due to limited permeability of the blood–brain barrier for dicarboxylic acids^44^, corresponding to the similarly high a priori risk of striatal damage in both HE and LE patients^2,3^. As an additional possible mechanism explaining differences between individuals with HE and LE subtype, selective glutarylation of more than 30 mitochondrial proteins exclusively localized in astroglial cells of Gcdh-deficient mice was recently demonstrated resulting in reduced catalytic activity, stability and protein–protein interaction of glutamate dehydrogenase and brain-specific carbonic anhydrase 5b. This process, in combination with elevated cerebral neurotoxic metabolites, may alter glial cell metabolism and coupling of astrocytes and neurons by harming anaplerotic transfers, increasing glutamate levels in the synaptic cleft, and affecting the formation of enzymatic supercomplexes which results in disturbed cerebral energy and neurotransmitter metabolism^45,46^. However, the specific dysfunctional effect of glutarylated proteins on mitochondrial function and its potential involvement in developing chronic and progredient whiter matter changes remains to be elucidated. It is also unclear whether increased cerebral concentrations of neurotoxic metabolites and progressive white matter abnormalities in HE patients are the cause of the poorer cognitive outcome in this study. Progression of white matter abnormalities might also affect cognition. Even if in this study no decline of cognitive performance over time was detected, extended long-term period remains unclear as this study covers only twenty years.

### Implications for long-term treatment?

In combination with ET, low lysine diet has been demonstrated to be an effective treatment for early diagnosed GA1 patients up to age six years, and previous studies including a recent meta-analysis with over 600 patients have demonstrated the positive effect of metabolic MT and ET on neurologic motor outcome with the vast majority of patients with full adherence to recommended therapy being prevented from striatal injury and remaining asymptomatic^1,8^. Other forms of dietary treatment or experimental therapeutic approaches in animal models, such as inhibition of upstream enzymes, have been less effective or failed to prevent the clinical phenotype^13,47–49^. Effectiveness of dietary treatment in patients beyond age six years receiving a liberalized protein-controlled diet is less clear, but a stable clinical and anthropometric long-term disease course up to adulthood has recently been reported^25,50^. However, prospective studies during the last decades have shown that the effect of dietary treatment in GA1 seems to be limited since (1) white matter changes are already found in patients identified by NBS while still following a low lysine diet, (2) MT may not stop or slow the progression of white matter changes^7^, and (3) chronic renal abnormalities are found in both HE and LE patients regardless the quality of therapy^1^. In line with this, our study did not detect an impact of therapy on cognitive outcome. Whether further restriction of lysine intake could prevent cognitive impairment in HE patients seems doubtful. Since lysine intake below the minimal requirement affects normal growth and neurological development and increases the risk of insidious-onset dystonia, a deviation from recommended dietary management should not be considered as safe^1,25^.

### Study limitations

Systematic and prospective evaluation of cognitive development is challenging in a rare metabolic disease such as GA1. However, our study population comprised the largest cohort worldwide that has been systematically analysed for cognitive functions in GA1. In addition, the study design included both, a cross-sectional analysis as well as a longitudinal design, which is considered the most high-quality design for developmental research. Nevertheless, we have to admit some limiting factors. First, we had to use a diversity of different neuropsychological tests including their revisions and adaptations of standard values as this study covers a long period of time of over 20 years. Second, the heterogeneous study cohort comprised different cultural backgrounds which may affect comprehensive performance in specific language-based but also non-verbal cognitive subtests. This may impact IQ test results that are standardized to a German reference population and may be a limitation in interpreting the data. Third, cognitive assessment of very young patients is generally difficult. And fourth, the limited number of patients with severe motor disabilities may result in a potential underestimation of the effect of severe MD on cognitive functions.

In summary, this study shows that neurologic impairment in GA1 is not only limited to motor deficits but is also related to cognitive functions. With HE patients being at risk for a poorer outcome, more effective long-term treatment concepts particularly for this subgroup are needed.

## Supplementary Information

Supplementary Information.

## Data Availability

The datasets of this study are not publicly available due to existing data protection laws. Data ownership is incumbent upon the members of the E-IMD consortium making data available for specific research purposes upon request.

## Abbreviations

ET: Emergency treatment
GA: Glutaric acid
GA1: Glutaric aciduria type 1
GCDH: Glutaryl-CoA dehydrogenase
HE: High excreter
LE: Low excreter
MD: Movement disorder
MT: Maintenance treatment
NBS: Newborn screening
