# Publisher Correction: The biochemical subtype is a predictor for cognitive function in glutaric aciduria type 1: a national prospective follow-up study

**DOI:** 10.1038/s41598-021-00137-5

**Published:** 2021-10-12

**Authors:** E. M. Charlotte Märtner, Eva Thimm, Philipp Guder, Katharina A. Schiergens, Frank Rutsch, Sylvia Roloff, Iris Marquardt, Anibh M. Das, Peter Freisinger, Sarah C. Grünert, Johannes Krämer, Matthias R. Baumgartner, Skadi Beblo, Claudia Haase, Andrea Dieckmann, Martin Lindner, Andrea Näke, Georg F. Hoffmann, Chris Mühlhausen, Magdalena Walter, Sven F. Garbade, Esther M. Maier, Stefan Kölker, Nikolas Boy

**Affiliations:** 1 Division of Child Neurology and Metabolic Medicine, Centre for Child and Adolescent Medicine, University Hospital Heidelberg, Heidelberg, Germany; 2 Division of Experimental Paediatrics and Metabolism, Department of General Paediatrics, Neonatology and Paediatric Cardiology, University Children’s Hospital, Heinrich Heine University Düsseldorf, Düsseldorf, Germany; 3 Children’s Hospital, University Medical Center Hamburg-Eppendorf, Hamburg, Germany; 4 Dr. Von Hauner Children’s Hospital, Ludwig-Maximilians-University, Munich, Germany; 5 Department of General Paediatrics, Metabolic Diseases, University Children’s Hospital Muenster, Muenster, Germany; 6 Charité—Universitätsmedizin Berlin, corporate member of Freie Universität Berlin, Humboldt-Universität Zu Berlin, and Berlin Institute of Health, Center for Chronically Sick Children, Berlin, Germany; 7 Department of Child Neurology, Children’s Hospital Oldenburg, Oldenburg, Germany; 8 Department of Paediatrics, Paediatric Metabolic Medicine, Hannover Medical School, Hannover, Germany; 9 Children’s Hospital Reutlingen, Reutlingen, Germany; 10 Department of General Paediatrics, Adolescent Medicine and Neonatology, Medical Centre, University of Freiburg, Faculty of Medicine, Freiburg, Germany; 11 Department of Pediatric Neurology and Inborn Errors of Metabolism, Children’s Hospital, University of Ulm, Ulm, Germany; 12 Division of Metabolism and Children’s Research Centre, University Children’s Hospital Zurich, Zurich, Switzerland; 13 Department of Women and Child Health, Hospital for Children and Adolescents, Centre for Paediatric Research Leipzig (CPL), University Hospitals, University of Leipzig, Leipzig, Germany; 14 Department of Pediatrics, Helios Klinikum, Erfurt, Germany; 15 Centre for Inborn Metabolic Disorders, Department of Neuropediatrics, Jena University Hospital, Jena, Germany; 16 Division of Paediatric Neurology, University Children’s Hospital Frankfurt, Frankfurt, Germany; 17 Children’s Hospital Carl Gustav Carus, Technical University, Dresden, Germany; 18 Department of Pediatrics and Adolescent Medicine, University Medical Center, Göttingen, Germany

Correction to: *Scientific Reports* 10.1038/s41598-021-98809-9, published online 29 September 2021

In the original version of this Article Nikolas Boy was incorrectly affiliated with ‘Division of Paediatric Neurology and Metabolic Medicine, Department of General Paediatrics, Centre for Paediatrics and Adolescent Medicine, University Hospital Heidelberg, 69120, Heidelberg, Germany’. The correct affiliation is listed below.

Division of Child Neurology and Metabolic Medicine, Centre for Child and Adolescent Medicine, University Hospital Heidelberg, Heidelberg, Germany.

The original Article has been corrected.

