# Supplementary material for: The biochemical subtype is a predictor for cognitive function in glutaric aciduria type 1: a national prospective follow-up study
Source: Sci Rep. 2021 Sep 29;11:19300. doi: 10.1038/s41598-021-98809-9 (PMC8481501; doi:10.1038/s41598-021-98809-9)
Supplement: Supplementary file 1 — Supplementary Information. [file 41598_2021_98809_MOESM1_ESM.pdf]

Supplement

Supplementary Table 1: Study population, individuals identified by NBS.

A; abnormal; ET, emergency treatment; F, Female; interm, Intermediate; HAWIVA, Hannover-Wechsler-Intelligenztest für das Vorschulalter; K-ABC, Kaufmann Assessment Battery for Children; M, Male; MT, maintenance treatment; N, normal; NBS, newborn screening; PID, Patient's identification; SON-R, Snijders-Oomen Nonverbal Intelligence Test Revised; WAIS, Wechsler Adult Intelligence Scale; WISC, Wechsler Intelligence Scale for Children; WPPSI, Wechsler Preschool and Primary Scale of Intelligence.

PID	Age at last test [years]	Sex	Biochemical subtype	MT	ET	Neurologic abnormality	Denver Developmental Screening Test					Bayley Scales of Infant Development	Intelligence tests								
														Intelligence test at last visit							
							Total	Personal-social	Fine motor and adaptive	Language	Gross motor	Cognitive Scale	Number of tests	Type of test	Full scale IQ	Fluid reasoning	Crystallised intelligence	Visual processing	Short-term memory	Processing speed
1	0.67	M	LE	No	Yes	None	N	N	N	N	N	-	-	-	-	-	-	-	-	-
2	0.95	F	HE	Yes	Yes	None	N	N	N	N	N	-	-	-	-	-	-	-	-	-
3	1.67	F	HE (interm)	No	No	Major	A	N	N	A	A	-	-	-	-	-	-	-	-	-
4	2.08	M	HE	Yes	Yes	None	N	N	N	N	N	-	-	-	-	-	-	-	-	-
5	2.09	F	HE (interm)	Yes	Yes	None	N	N	N	N	N	-	-	-	-	-	-	-	-	-
6	2.1	M	HE	No	No	Major	-	-	-	-	-	49	-	-	-	-	-	-	-	-
7	2.26	F	HE	Yes	Yes	None	N	N	N	N	N	-	-	-	-	-	-	-	-	-
8	2.51	F	HE	Yes	Yes	None	N	N	N	N	N	-	-	-	-	-	-	-	-	-
9	3	F	HE	Yes	Yes	None	-	-	-	-	-	91	-	-	-	-	-	-	-	-
10	3.36	M	HE	No	No	Minor	-	-	-	-	-	-	2	WPPSI-III	101	-	104	-	-	-
11	3.36	M	HE	No	No	Major	A	N	A	N	A	-	-	-	-	-	-	-	-	-
12	3.71	F	LE	No	Yes	Major	-	-	-	-	-	110	-	-	-	-	-	-	-	-

13	3.76	F	HE	Yes	Yes	None	-	-	-	-	-	-	1	WPPSI-III	-	-	-	90	-	-
14	3.85	M	LE	Yes	Yes	None	-	-	-	-	-	-	2	WPPSI-III	86	-	100	-	-	-
15	3.99	M	HE	Yes	Yes	None	N	N	N	N	N	-	-	-	-	-	-	-	-	-
16	4.45	F	HE	Yes	Yes	Minor	-	-	-	-	-	110	1	WPPSI-III	98	94	96	-	-	91
17	4.56	M	HE	No	Yes	None	-	-	-	-	-	-	1	WPPSI-III	81	88	88	88	-	60
18	4.6	F	-	Yes	Yes	None	-	-	-	-	-	-	1	WPPSI-III	80	84	75	82	-	83
19	4.95	M	HE	No	Yes	Major	-	-	-	-	-	-	1	WPPSI-III	91	84	93	84	-	91
20	5.22	F	HE	Yes	Yes	None	-	-	-	-	-	86	2	HAWIVA-III	88	95	89	95	-	-
21	5.24	F	LE	Yes	Yes	None	N	N	N	N	N	116	2	WPPSI-IV	104	-	93	-	-	-
22	5.39	M	HE	Yes	Yes	Minor	-	-	-	-	-	-	1	WPPSI-III	80	86	78	86	-	71
23	5.42	F	HE	Yes	Yes	Major	A	A	A	A	A	93	-	-	-	-	-	-	-	-
24	5.52	M	LE	Yes	Yes	None	-	-	-	-	-	-	1	WPPSI-III	93	96	100	96	-	83
25	5.68	M	HE	Yes	Yes	None	-	-	-	-	-	-	1	WPPSI-III	103	114	100	114	-	97
26	5.94	F	-	Yes	Yes	None	-	-	-	-	-	-	1	WPPSI-III	77	82	75	82	-	83
27	6.02	M	LE	Yes	Yes	None	-	-	-	-	-	103	1	HAWIVA-III	101	104	101	104	-	103
28	6.08	F	HE	Yes	Yes	None	-	-	-	-	-	-	3	SON-R 2,5-7	82	-	-	-	-	-
29	6.31	F	LE	Yes	No	Major	-	-	-	-	-	-	2	SON-R 2,5-7	61	75	-	57	-	-
30	6.54	M	LE	Yes	Yes	None	N	N	N	N	N	107	3	WPPSI-III	99	96	112	96	-	78
31	6.93	F	HE	Yes	Yes	None	N	N	N	N	N	97	4	WPPSI-III	81	86	88	86	-	73
32	7.05	F	HE	Yes	Yes	None	N	N	N	N	N	95	2	WISC-V	64	64	68	75	67	69
33	7.13	M	HE (interm)	No	Yes	Major	-	-	-	-	-	81	2	K-ABC	59	-	-	-	-	-
34	7.56	M	HE	Yes	Yes	None	N	N	N	N	N	-	1	WISC-V	73	74	84	78	62	100
35	7.82	M	HE	Yes	Yes	Major	A	N	A	N	A	-	1	WISC-V	94	94	106	97	76	-
36	7.93	M	HE	No	Yes	Major	A	A	N	N	A	74	1	WISC-IV	84	86	90	86	93	81
37	7.95	M	HE	No	Yes	Minor	-	-	-	-	-	-	1	K-ABC	62	-	-	-	-	-
38	8.1	M	HE	Yes	Yes	Minor	-	-	-	-	-	-	1	WISC-V	58	64	59	69	-	89
39	8.75	F	HE	No	Yes	Major	-	-	-	-	-	-	1	WISC-V	63	58	86	69	62	80
40	8.94	F	HE (interm)	Yes	Yes	None	-	-	-	-	-	-	1	WISC-V	85	79	89	86	85	105
41	9.11	M	HE (interm)	No	Yes	Major	-	-	-	-	-	-	1	WISC-IV	112	112	119	112	117	86
42	10.44	F	HE	No	Yes	Major	A	A	A	A	A	-	-	-	-	-	-	-	-	-
43	10.93	F	LE	Yes	Yes	None	-	-	-	-	-	-	1	WISC-V	113	126	95	97	103	119

44	10.99	F	LE	Yes	Yes	None	-	-	-	-	-	-	1	WISC-V	87	85	81	-	-	103
45	11.22	F	HE (interm)	Yes	Yes	None	-	-	-	-	-	120	1	WISC-IV	106	100	109	100	108	103
46	11.34	F	LE	Yes	Yes	None	-	-	-	-	-	-	1	K-ABC II	97	91	100	103	91	-
47	11.69	F	HE	Yes	Yes	None	N	N	N	N	N	-	1	WISC-V	99	94	100	102	107	92
48	11.76	M	HE	Yes	Yes	None	-	-	-	-	-	-	2	WISC-V	94	118	81	-	-	89
49	11.91	F	HE	Yes	Yes	None	-	-	-	-	-	-	3	WISC-IV	88	102	85	102	96	83
50	11.96	M	HE (interm)	No	No	Major	-	-	-	-	-	-	1	WISC-V	97	103	92	105	97	105
51	11.98	F	HE	Yes	Yes	Major	-	-	-	-	-	62	4	WISC-IV	97	100	97	100	105	88
52	12.03	M	HE (interm)	Yes	Yes	Major	-	-	-	-	-	-	1	WISC-V	99	115	84	117	107	98
53	12.09	F	LE	Yes	Yes	None	N	N	N	N	N	94	5	WISC-V	107	106	95	117	100	116
54	12.35	M	HE	Yes	Yes	Minor	N	N	N	A	N	-	1	WISC-V	69	76	73	75	62	83
55	12.56	F	HE	Yes	Yes	None	-	-	-	-	-	86	2	WISC-IV	109	112	117	112	96	97
56	13.53	M	HE (interm)	Yes	Yes	None	-	-	-	-	-	71	1	WISC-IV	102	112	111	112	105	100
57	13.86	M	HE	Yes	No	Major	-	-	-	-	-	-	1	WISC-V	71	74	89	-	-	-
58	14.13	M	HE	No	Yes	None	-	-	-	-	-	-	1	WISC-V	76	88	81	64	76	89
59	15.5	M	HE	Yes	Yes	None	-	-	-	-	-	-	3	WISC-V	82	94	86	81	88	89
60	15.86	F	HE	Yes	Yes	Minor	-	-	-	-	-	-	3	WISC-IV	78	-	97	-	-	-
61	15.93	F	LE	No	Yes	Major	N	N	N	N	A	-	2	WISC-V	95	106	106	84	100	95
62	16.08	F	HE	Yes	Yes	None	-	-	-	-	-	-	1	WISC-V	60	64	59	72	-	72
63	16.15	F	HE	Yes	Yes	None	-	-	N	A	N	-	1	WISC-V	79	94	78	92	79	100
64	16.37	F	HE	Yes	Yes	None	-	-	-	-	-	-	5	WISC-V	83	79	84	-	-	-
65	16.96	F	HE	No	Yes	None	-	-	-	-	-	-	1	WISC-V	75	79	78	86	76	83
66	16.98	F	LE	Yes	Yes	None	-	-	-	-	-	-	2	WISC-V	108	109	92	92	120	123
67	17.31	M	HE	Yes	No	Major	-	-	-	-	-	-	5	WAIS-IV	67	65	74	65	77	73
68	17.77	M	HE	Yes	Yes	None	-	-	-	-	-	-	2	WAIS-IV	94	91	88	91	102	106
69	17.89	F	HE	Yes	Yes	Major	-	-	-	-	-	-	1	WAIS-IV	-	52	-	52	-	-
70	19.01	M	HE (interm)	Yes	Yes	None	N	N	N	N	N	91	4	WAIS-IV	86	83	88	83	102	83
71	19.05	M	HE (interm)	Yes	No	Major	-	-	-	-	-	-	2	WAIS-IV	83	98	84	98	82	79
72	19.07	F	HE	No	Yes	Major	-	-	-	-	-	-	1	WAIS-IV	89	96	96	96	82	91
